# Critically appraised topic on adverse food reactions of companion animals (6): prevalence of noncutaneous manifestations of adverse food reactions in dogs and cats

**DOI:** 10.1186/s12917-018-1656-0

**Published:** 2018-11-12

**Authors:** Ralf S. Mueller, Thierry Olivry

**Affiliations:** 1 Medizinische Kleintierklinik, Centre for Clinical Veterinary Medicine, LMU Munich, Veterinaerstrasse 13, 80539 Munich, Germany; 2 Department of Clinical Sciences, College of Veterinary Medicine, North Carolina State University, 1060 William Moore Drive, Raleigh, NC 27607 USA

**Keywords:** Canine, Feline, Food allergy, Gastroenteritis, Vomiting, Diarrhoea

## Abstract

**Background:**

Many reports describe the cutaneous signs of adverse food reactions (AFR) in the dog and cat. However, various non-cutaneous clinical signs are less well described. Our objective was to systematically evaluate these non-cutaneous signs of AFR in small animals.

**Results:**

We searched two databases (MEDLINE and Web of Science) for pertinent references on non-cutaneous signs of adverse food reactions. Among 117 and 764 articles found in the MEDLINE and Web of Science databases, respectively, we selected 47 articles that reported data related to non-cutaneous clinical signs of AFR. Gastrointestinal signs, symmetrical lupoid onychitis, conjunctivitis, sneezing, and anaphylaxis were reported to be associated with AFR in dogs and gastrointestinal and respiratory signs, conjunctivitis, and hyperactive behaviour in cats. In Border terriers with paroxysmal gluten-sensitive dyskinesia, an underlying AFR should be considered. Of these clinical signs diarrhoea and frequent defecation were most frequently reported to be diet-responsive in dogs; in the cat, these were vomiting and diarrhoea.

**Conclusions:**

An elimination diet should be considered early in the work-up of dogs and cats with chronic vomiting and diarrhoea. Other non-cutaneous signs occur less commonly because of AFRs.

## Background

Atopic dermatitis and urticaria are well-recognised clinical features of adverse food reaction (AFR) in the dog [1, 2]. Cutaneous reaction patterns such as miliary dermatitis, variants of the so-called “eosinophilic granuloma complex” (including oral lesions [3]) and non-inflammatory alopecia have been reported with AFR in cats [4–7]. Non-cutaneous clinical signs are mainly gastrointestinal, but a systematic review of reported non-cutaneous clinical signs is lacking. Our objective was to systematically evaluate those non-cutaneous signs of AFR in dogs and cats.

### Clinical scenario

Consider the example of two patients: A 15-month-old castrated male Jack Russell terrier with chronic diarrhoea and concurrent bilateral conjunctivitis and a six-year-old female spayed domestic shorthaired cat with flatulence and frequent vomiting. You inform the owners of both patients that you suspect that their clinical signs might be caused by a reaction to a component of their pet’s diet and you advise that an elimination diet with ingredients not previously fed is indicated for 8 weeks to evaluate a potential food involvement [8]. The owners ask you what clinical signs besides itching and skin problems are frequently reported to be caused by an AFR.

### Structured question

*Which are the non-cutaneous clinical signs of AFR reported in dogs and cats and how often do they occur?*

### Search strategy

We searched the Web of Science (Core Collection) and MEDLINE databases using the following string: ((dog* or canine or cat* or feline) and (food* or diet*) and (allerg* or hypersens*)) not (human* or child* or adult*). We limited the search to journal articles published from 1980 to 2017; there were no language restrictions. Bibliographies from selected articles and proceedings of recent conferences in veterinary dermatology and internal medicine were also searched.

### Identified evidence

Our literature search identified 117 and 764 articles in the MEDLINE and Web of Science (Core Collection) databases, respectively. Abstracts of relevant titles were screened and any potentially useful manuscript was downloaded and read. The bibliography of these articles was examined further for additional pertinent citations. In addition, proceedings of recent veterinary dermatology or internal medicine conferences were perused. Altogether, we selected 47 papers that provided usable information.

### Evaluation of evidence

In prospective studies, improvement of a clinical sign with an elimination diet, recurrence after a re-challenge with the previous diet and repeated improvement again when feeding the diet were considered to be strong evidence for an AFR causing that clinical sign. If those conditions were met, but the study was retrospective, we considered the evidence to be only of moderate strength. When improvement occurred upon a change in diet, but a re-challenge with the previous food was not performed, then the evidence was considered weak. The prospective or retrospective nature of some studies was unclear, and we deemed the evidence provided by those reports as of moderate strength. The non-cutaneous clinical signs of AFR, the number of animals affected, as well as the strength of evidence are listed in Table 1 for dogs and in Table 2 for cats.

**Table 1:** Non-cutaneous clinical signs of AFR in dogs

Clinical sign	Number of animals (% of all reported non-cutaneous AFR)	Strength of evidence
Diarrhoea [9–19, 22–27, 32–44, 47, 48]	391–490^a^ (70–88%)	Strong in 36–39^a^ [9–19, 48] Moderate in 151–189^a^ [22–27, 47] Weak in 204–212^a^ [32–44]
Vomiting [9, 11, 13–15, 17, 18, 22–27, 33, 34, 40, 42, 43]	28–115^a^ (5–21%)	Strong in 6–47^a^ [9, 11, 13–15, 17, 18] Moderate in 5–43^a^ [22–27] Weak in 17–25 [33, 34, 40, 42, 43]
Increased frequency of defecation [23, 24, 45]	33 (6%)	Strong in 16 [45] Moderate in 17 [23, 24]
Tenesmus [45]	11 (2%)	Strong in 11
Paroxysmal gluten-sensitive dyskinesia of Border terriers [49]	5 (1%)	Strong in 2 Weak in 3
Symmetrical lupoid onychodystrophy [50]	4 (1%)	Strong in 2 Weak in 2
Anaphylaxis [40]	1 (0.2%)	Weak
Conjunctivitis [12]	1 (0.2%)	Strong
Asthma [27]	1 (0.2%)	Moderate
Sneezing [14]	1 (0.2%)	Strong

^a^ Minimal and maximal number of dogs, as in some studies, the specific number of dogs showing some of the individual clinical signs was not reported

**Table 2:** Non-cutaneous clinical signs of AFR in cats

Clinical sign	Number of animals (% of all reported non-cutaneous AFR)	Strength of evidence
Diarrhoea [5, 7, 20, 21, 27–31, 51]	25–49^a^ (28–55%)	Strong in 11–16 [20, 21] Moderate in 14–33 [5, 7, 27–31] Weak in 1 [51]
Vomiting [4, 5, 7, 20, 21, 27–29, 31]	26–46^a^ (29–52%)	Strong in 15–20 [20, 21] Moderate in 8–27 [4, 5, 7, 28, 29, 31, 51]
Conjunctivitis [4, 7, 46]	3–20^a^ (3–22%)	Strong in 1 [7] Moderate in 1 [4] Weak in 1–18 [46]
Salivating [46]	1–18 (1–20%)	Weak
Respiratory signs [7]	4 (4%)	Moderate
Flatulence [5]	3 (3%)	Moderate
Hyperactive behaviour [6]	1 (1%)	Strong

^a^ Minimal and maximal number of cats, as in some studies, the specific number of cats showing some of the individual clinical signs was not reported

A high number of dogs and cats with AFR were reported to exhibit vomiting and/or diarrhoea. Although, in many publications, the evidence for a causative AFR was strong [9–21], some cohorts were only retrospective studies [4, 5, 22–31]. Finally, in others groups of animals, the diagnosis of AFR was solely based on an improvement with diet change without report of a re-challenge with the previously fed diet [32–44]. Unfortunately, in a number of articles, these two clinical signs were not listed separately, and patients were reported with “vomiting or diarrhoea” or “gastrointestinal signs” without specifying the number of animals having exhibited each individual sign [7, 9, 14, 15, 17, 24, 25, 28]. Similarly, defecation and tenesmus in dogs [45], and conjunctivitis and drooling in cats [46] were not always clearly distinguished. Of the 395 dogs in which vomiting and diarrhoea were reported individually, 368 dogs (93%) had diarrhoea, six had vomiting (2%) and in 21 dogs (5%) both clinical signs were present [10, 11, 13, 16, 18, 19, 22, 32, 36, 38–42, 44, 47]. Of the 40 cats with individually reported vomiting and diarrhoea, 15 vomited (38%), 18 had diarrhoea (45%) and seven (18%) were reported with both clinical signs [5, 20, 21, 27, 29]. These numbers suggest that AFR-associated vomiting is more prevalent in cats than in dogs. When comparing cutaneous with gastrointestinal signs in dogs and cats (where possible), a bias from authors was apparent. Cutaneous signs predominated when the study had been performed by dermatologists, where 261 dogs (71%) and 89 cats (77%) showed cutaneous signs only, ten dogs (3%) and two cats (2%) showed only gastrointestinal signs and 97 dogs (27%) and 23 cats (20%) showed both gastrointestinal and cutaneous signs. When the studies where performed by internists, 94 dogs (73%) and 19 cats (49%) showed gastrointestinal signs only, ten dogs (8%) and ten cats (26%) showed cutaneous signs only, and 24 dogs (19%) and ten cats (26%) showed both.

## Conclusion and implication for practitioners

We evaluated 34 articles reporting dogs with non-cutaneous clinical signs, 16 of those provided strong evidence, six moderate evidence and 12 weak evidence. In the cat, strong evidence was provided by only three reports, moderate by seven and weak evidence by two. One publication of moderate quality reported on both dogs and cats. Vomiting and diarrhoea were the non-cutaneous signs reported in more than 20% of dogs and cats with AFR, and an elimination diet with subsequent re-challenge is indicated for those animals early in the diagnostic work-up. In dogs, anaphylaxis, conjunctivitis, increased frequency of defecation, symmetric lupoid onychitis, and sneezing were reported less commonly. In cats, uncommon noncutaneous signs of AFR were conjunctivitis, salivating, flatulence, hyperactive behaviour and respiratory signs and all of these were more often associated with diseases other than an AFR. Depending on the patient’s history, other tests should possibly be conducted before an elimination diet or concurrently. The probability of AFR is likely to increase if patients exhibit more than one of the non-cutaneous signs described above.

## Abbreviations

AFR(s): Adverse food reaction(s)
CAT: Critically appraised topic
